# Social Fear Conditioning Paradigm in Virtual Reality: Social vs. Electrical Aversive Conditioning

**DOI:** 10.3389/fpsyg.2017.01979

**Published:** 2017-11-14

**Authors:** Jonas Reichenberger, Sonja Porsch, Jasmin Wittmann, Verena Zimmermann, Youssef Shiban

**Affiliations:** Department of Clinical Psychology and Psychotherapy, Institute of Psychology, University of Regensburg, Regensburg, Germany

**Keywords:** social fear conditioning, virtual reality, fear-potentiated startle, skin conductance level, avoidance behavior

## Abstract

In a previous study we could show that social fear can be induced and extinguished using virtual reality (VR). In the present study, we aimed to investigate the belongingness effect in an operant social fear conditioning (SFC) paradigm which consisted of an acquisition and an extinction phase. Forty-three participants used a joystick to approach different virtual male agents that served as conditioned stimuli. Participants were randomly allocated to one of two experimental conditions. In the electroshock condition, the unconditioned stimulus (US) used during acquisition was an electric stimulation. In the social threat condition, the US consisted of an offense: a spit in the face, mimicked by a sound and a weak air blast to the participant’s neck combined with an insult. In both groups the US was presented when participants were close to the agent (75% contingency for CS+). Outcome variables included subjective, psychophysiological and behavioral data. As expected, fear and contingency ratings increased significantly during acquisition and the differentiation between CS+ and CS- vanished during extinction. Furthermore, a clear difference in skin conductance between CS+ and CS- at the beginning of the acquisition indicated that SFC had been successful. However, a fast habituation to the US was found toward the end of the acquisition phase for the physiological response. Furthermore, participants showed avoidance behavior toward CS+ in both conditions. The results show that social fear can successfully be induced and extinguished in VR in a human sample. Thus, our paradigm can help to gain insight into learning and unlearning of social fear. Regarding the belongingness effect, the social threat condition benefits from a better differentiation between the aversive and the non-aversive stimuli. As next step we suggest comparing social-phobic patients to healthy controls in order to investigate possible differences in discrimination learning and to foster the development of more efficient treatments for social phobia.

## Introduction

Social anxiety disorder (SAD) is one of the most relevant anxiety disorders. It is characterized by intense anxiety when faced with social interactions along with physical symptoms like blushing or trembling, and extreme avoidance behavior concerning social interaction (Fehm et al., 2005; Kessler et al., 2005; American Psychiatric Association, 2013). While learning models are relatively well established in specific phobia, PTSD and panic disorders, learning paradigms for SAD are far less developed, both in animal models and in humans. Besides the diathesis stress model, there is evidence showing that fear conditioning may play an essential role in the development and maintenance of SAD (Mineka and Zinbarg, 2006; Mineka and Oehlberg, 2008).

Cognitive-behavioral therapy is the method of choice for the treatment of SAD; it is widely supported by current research and therefore assumed to be a reliable approach for overcoming anxiety (Arch et al., 2012). Cognitive-behavioral therapy is also often combined with exposure to feared situations in order to maximize the therapeutic success (Wolitzky-Taylor et al., 2008). Nevertheless, the effectiveness of this treatment approach is not always satisfactory and a high number of non-responders remain (Norton and Price, 2007).

Empirical findings show that conditioning mechanisms play an important role in the etiology of the elementary processes of SAD, making them essential to examine in order to maximize the impact of psychotherapeutic interventions (Mineka and Zinbarg, 2006; Mineka and Oehlberg, 2008). Classical fear conditioning (according to Pavlov) is a form of associative learning in which an organism learns to associate two stimuli with each other (Pavlov, 1927). E.g., hearing someone laugh (unconditioned stimulus: US) while giving a speech may result in the speaker showing a fear response (unconditioned response: UR). As a result, the previously neutral stimulus (giving a speech), now called conditioned stimulus (CS), triggers the newly learned fear reaction (conditioned response: CR). Classical fear conditioning is considered a central pathogenic pathway in anxiety disorders (Lissek et al., 2005; Mineka and Zinbarg, 2006; LeDoux, 2014). Operant fear conditioning (learning by consequences) may be also be relevant for the development of anxiety disorders, because it relates to stimuli that reinforced or punished the person during approach behavior. E.g., if voluntarily presenting a paper is followed by the lecturer harshly criticizing the presentation, a student might no longer report voluntarily in the future. Thus, other persons and social interactions might be prototypical stimuli involved in operant learning processes. However, until now little research has been conducted on operant fear conditioning in SAD.

Fear conditioning in mice in social as well as non-social contexts is addressed in the social fear conditioning (SFC) approach investigated by Toth et al. (2012). In this paradigm, naturally occurring preference behavior of male rodents toward an unknown conspecific was paired with an aversive US, namely an electric stimulus applied to the foot for 1 s. During acquisition phase, the rodents learned to associate the appearance of the negative stimulus with the conspecific, which induced social fear including avoidance behavior. In a following extinction phase on the next day, different male conspecifics were presented to the experimental animals in their cage without any negative US. It could be observed that avoidance and fear-driven behavior were extinguished and replaced by the naturally occurring preference behavior again. Therefore, in the course of the experiment, acquisition and extinction of fear were demonstrated. These results suggest that, using the applied paradigm, it is possible to draw conclusions about the etiology of SAD and potential leverage points for future treatment approaches (Toth et al., 2012; Toth and Neumann, 2013; Zoicas et al., 2014).

Many uncontrollable contextual and environmental factors can play a role and therefore turn out to be confounding variables in human experimental as well as therapeutic settings. A way to circumvent this problem is conducting experiments in virtual reality (VR), which also allows for the creation of paradigms of SAD development and the exploration of potential treatment improvements. The use of an artificially designed virtual environment minimizes potentially confounding variables by presenting standardized situations to participants. Subjects are able to interact with their environment and diverse stimuli can be applied in a multimodal manner (Bohil et al., 2011). Furthermore, it is possible to directly record the participant’s reactions to the stimuli in the form of verbal ratings, fear-potentiated startle or electrocardiographic data (Mühlberger et al., 2007). Thus, VR allows conducting SFC related experiments in a realistic, standardized environment in an economic and easily administrable manner. An additional advantage of VR that is particularly important in the treatment of SAD is the prevention of avoidance behavior, which often leads to the reinforcement of anxiety symptoms (American Psychiatric Association, 2013). In general, the results of conditioning processes in VR are hugely satisfying (Huff et al., 2010).

Shiban et al. (2015) implemented a procedure similar to the SFC paradigm designed for mice by Toth et al. (2012) in order to investigate SFC in humans in VR. In this experimental setting, participants had to actively approach different agents in VR using a joystick. During the acquisition phase, one of the agents, referred to as CS+, was paired with an US, a loud female scream combined with an air blast. During the extinction and the following generalization test phase, no US was administered. In line with the initial hypotheses, participants rated the CS+ as significantly less pleasant than the CS- after the acquisition phase. These results were also supported by the heart rate pattern, as the heart rate was higher for the CS+ than for the CS- after acquisition. After the extinction phase, the ratings returned to an equal level and the fear-potentiated startle response decreased. Interestingly, during the generalization test, the more socially fearful participants rated every agent as less pleasant, compared to the less socially fearful participants who only rated the CS+ as less pleasant. This indicates that more socially fearful participants tend to generalize the unpleasantness of social stimuli to a broader context. In sum, SFC could be induced and extinguished successfully, thus emphasizing the role of operant conditioning in social fear learning. Nonetheless, the study has some limitations, which could be addressed in order to potentially improve the paradigm.

For instance, it is possible to manipulate the intensity of the social contact between the agent and the participant to investigate the specificity of the paradigm for social situations. We believe that our paradigm provides the opportunity for basic social interaction between the agent and the participant (via eye contact, self-regulated movement of the avatar and movement toward the agent). In the current study we improved upon this aspect by designing a social threat condition and comparing it to a conventional electroshock condition. Furthermore, it could be criticized that the amount of social interaction in the preliminary study was quite low, as the agent did not directly communicate with the participant. This has been taken into account in the current study, as in the social threat condition the agent verbally insults and spits at the participant is much more ecologically valid than the mere administration of an air blast or an electrical stimulation. We assume that it enables us to better use the paradigm for social fear research. In addition, the facial expressions of the agents were adjusted to the verbal utterance in order to create a more realistic and therefore more threatening experience. This also provided the opportunity to investigate the belongingness effect, since the accordance between the US and the CS plays an important role in conditioning. This concept was investigated in a study conducted by Hamm et al. (1989), in which pairs of unconditioned and neutral stimuli were rated according to their belongingness. After a classical conditioning process using rating-defined high- and low-belongingness pairs, finger pulse responses revealed significantly stronger acquisition and resistance to extinction for high-belongingness pairs.

Our current study is a further investigation of the SFC paradigm in VR in a human sample using an operant conditioning setting, which consisted of acquisition and extinction phases similar to those in the preliminary study. In the current study, we tried to maximize the immersion in VR using a head-mounted-display with a larger field of view as suggested in our first SFC study (Shiban et al., 2015). During the SFC process, fear and contingency ratings as well as physiological (fear-potentiated startle and skin conductance level) and behavioral data were collected. In order to take the above-mentioned effects of belongingness into account, a second experimental condition was added to the previous design. Besides the electroshock condition, in which an electrical stimulation to the lower arm serves as an US, an air blast combined with virtual spitting and insulting was employed as the US in the social threat condition. Because the subjective experience of (un)pleasantness was only partly in accordance with the physiological measurements in our first SFC study, we decided to use the skin conductance level (SCL) as an additional measure of distress during social interaction (e.g., Mesa et al., 2014). Moreover, we investigated the avoidance behavior quantified as the time in non-motion before the approach as well as the time in motion of the approach.

In our current study, we expected that (1) in the operant conditioning process, fear and contingency ratings for CS+ would increase after the acquisition phase compared to the baseline phase. Furthermore, (2) the amplitude of the fear-potentiated startle and the SCL as well as the time in non-motion before approaching the CS+ and time in motion of the approach toward CS+ were expected to increase. (3) After the extinction phase, fear and contingency ratings of the CS+ were supposed to return to baseline levels along with the electrophysiological reactions and the behavioral variables. (4) For the CS- and neutral stimulus (NS), no such changes were expected, i.e., the ratings and physiological measurements should remain stable. (5) The acquisition and the resistance to extinction were expected to be higher for the social threat condition than for the electroshock condition due to the belongingness effect of spitting and insulting to socially frightening situations and thus the more realistic simulation of social interaction. Finally, (6) a stronger manifestation of the conditioning process was expected in more socially fearful participants in comparison to less socially fearful participants.

## Materials and Methods

### Participants

Forty-four healthy volunteers were recruited through advertisements at the University of Regensburg. Exclusion criteria were age below 18 or above 55, a current diagnosis of psychiatric disorder, psychological treatment, history of psychotropic drug use, color blindness and uncorrected vision or hearing deficits. These criteria were assessed via a questionnaire after written informed consent had been obtained. Participants were randomly allocated to one of the two conditions. As one participant was excluded due to a technical error during data acquisition, the study comprised a total of forty-three participants (22 participants in the electroshock condition: 68.2% female, aged between 18 and 25, *M* = 21.10, *SD* = 1.80; and 21 participants in the social threat condition: 81% female, aged between 19 and 30, *M* = 21.95, *SD* = 2.84). All of the volunteers were students at the University of Regensburg and were offered credit points as compensation for their participation (see **Table 1**). The Ethics Committee of the University of Regensburg approved the study.

**Table 1:** Demographic variables and questionnaire data.

	Electric shock condition		Social threat condition			
	(*n* = 22)		(*n* = 21)				
Demographics	*M*	*SD*	*M*	*SD*	*df*	*t*	*p*
Age	21.10	1.80	21.95	2.84	41	-1.195	0.239
SPIN	12.95	9.23	16.67	11.71	41	-1.157	0.254
	***n***	***%***	***n***	***%***	***df***	**χ^2^**	***p^a^***
Gender *[female]*	15	68.2	17	81.0	1	0.920	0.337

*Means (*M*) and Standard Deviations (*SD*) and also *t*- and *p*-values are given for all participants for the variables age and SPIN (German version of the Social Phobia Inventory by Stangier and Steffens, 2002). Numbers of participants (*n*) and percent (*%*) for gender is given; ^a^Chi square test, two-tailed.*

### Apparatus

**FIGURE 1:**
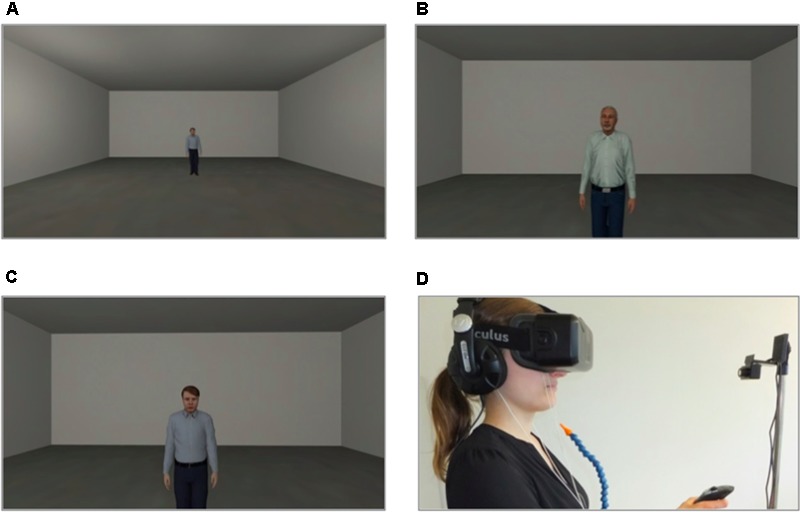
Virtual environment. **(A)** Room where all three phases took place. **(B,C)** Social stimuli (agents) used for the conditioning. **(D)** Setting (VR was presented via a head-mounted display) during the experiment (laboratory room was darkened).

The VR environment consisted of one room (see **Figure 1A**), in which all three phases (baseline, acquisition and extinction) took place. In every phase the participant was positioned at one end of the room and could see the agent at the opposite end of the room. The agents gazed dynamically at the participant and moved their head and upper body slightly (see **Figures 1B,C**). In 75% of the conditioning trials an aversive consequence followed when the participant reached the agent. Aversive consequences consisted of an electric stimulus to the participant’s lower arm in the electroshock condition or of an air blast to the right side of the participant’s neck (2 bar, 10 ms) accompanied by a sound of spitting followed by an insult in the social threat condition. In addition, when the participant approached the agent a startle sound was administered with a contingency of 75% in all phases. A compressed air tank was regulated via a magnetic valve system channeled the air blast through a tube that was fixed to the participant’s torso. A cuff was fixed to the participant’s right lower arm to administer the electric stimulus. Each participant’s individual pain threshold (*M* = 2.42 mA, *SD* = 1.82 mA) was determined before the VR session started. To this end, different strengths of electrical current were administered to the participant’s lower arm and then rated on a pain scale from 0 to 10. The amperage with a mean rating of 5 was used as the US during the VR session. The VR was presented to participants via an Oculus Rift DK2 head-mounted display (HMD; Oculus VR Inc., Irvine, CA, United States; see **Figure 1D**) and was generated via Steam Source engine (Valve Corporation, Bellevue, WA, United States). The presented VR environment was controlled by “cybersession” software (VTplus GmbH, Würzburg, Germany) (see **Figure 1C**). The participant’s head position was monitored via the Oculus’ electromagnetic tracking device (Oculus VR Inc., Irvine, CA, United States), which adjusts the field of view to any head movements. Sounds were presented over headphones (Sennheiser HD-215, Sennheiser electronic GmbH, Germany). Participants used a joystick (Logitech Extreme 3D Pro Joystick, Logitech GmbH, Germany) to move in the VR environment. Physiological data were monitored, digitally amplified (V-Amp, Brain Products GmbH, Germany) and recorded (Brain Vision Recorder software, Version 1.20, Brain Products GmbH, Germany).

### Measures

Participants filled out a demographic questionnaire (age, sex, education, and current occupation) and the Social Phobia Inventory (SPIN; Connor et al., 2000; German Version: Stangier and Steffens, 2002) to assess social fear.

The SPIN consists of 17 items that assess fear, avoidance, and physiological symptoms of social phobia in the previous week. Answers are given on a five-point Likert scale (from 0 = “not at all” to 4 = “extremely”). The German version of the SPIN was evaluated by Sosic et al. (2008). Internal consistency was excellent for a representative sample of 2043 Germans (Cronbach’s Alpha = 0.95). Convergent and divergent validity are satisfactory. Furthermore, the German version of the SPIN is a sensitive and specific measure for social phobia as it distinguishes successfully between social phobia and other psychiatric disorders (Sosic et al., 2008).

In order to measure the experienced fear and contingency of the agents, ratings were assessed verbally during the presentations of the agents in the rating phase following each of the three phases (“Estimate your fear now”; “How likely would an aversive stimulus have been?”). These ratings had a range from 0 (very low fear/very unlikely) to 100 (very high fear/very likely).

Besides the subjective measures, physiological data were collected. To record the electromyography of the musculus orbicularis oculi as a measure of fear-potentiated startle, four surface electrodes (Ag/AgCl, Ø = 8 mm) were affixed under the right eye of the participant and on the mastoid bones as reference and ground electrodes. Two additional surface electrodes (Ag/AgCl, Ø = 8 mm) were placed on the base of the thumb on the radial side of the palm of the non-dominant hand in order to record the SCL. The avoidance was measured as the time in non-motion (in s) before approaching the agents and the time in motion (in s) of the approach.

### Procedure

**FIGURE 2:**
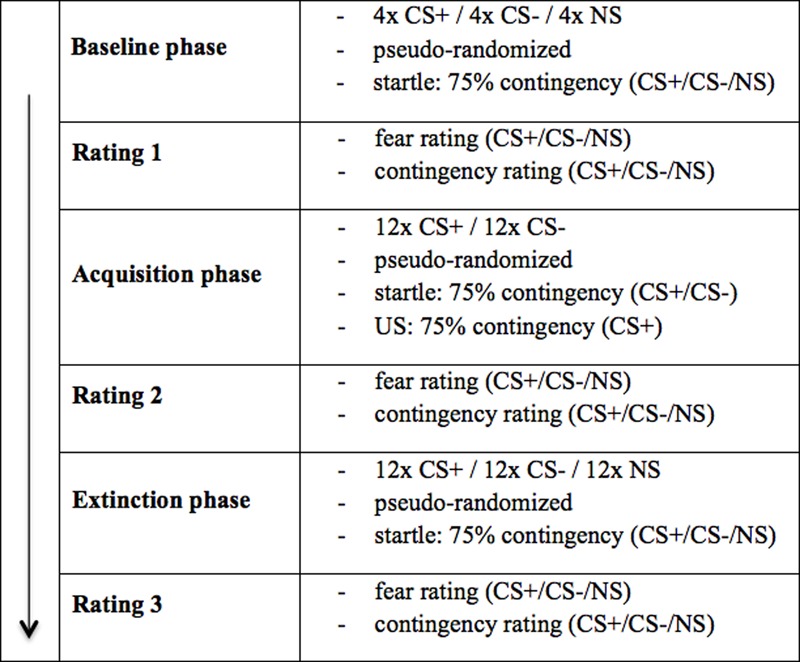
Experimental procedure. The experimental procedure took place as described above. As unconditioned stimulus (US), electrical stimulation (electro shock condition) or an air blast combined with virtual spitting and insulting (social threat condition) were applied. CS+ = agent paired with aversive US; CS– = agent without aversive US; *NS* = agent without aversive US and not appearing during the acquisition phase.

The experiment consisted of the questionnaire phase, the baseline phase, the acquisition phase and the extinction phase [total duration was 60 min (30 min in VR); see **Figure 2**].

The baseline phase consisted of four blocks. One block consisted of three presentations of each agent (CS+, CS-, NS), resulting in a total of 12 presentations of each agent per participant. The order within each block was randomized and no US was administered. Which agent was presented as CS+/CS-/NS, was balanced across participants. A startle noise (white noise: 103 dB, 10 ms) was presented with a contingency of 75%.

Conditioning was conducted in 12 blocks. One block consisted of two presentations of both conditioned stimuli with aversive reinforcement in terms of electric stimulus or air blast combined with virtual spitting and the negative utterance “Get lost!” (CS+) and without aversive reinforcement (CS-), resulting in a total of 24 presentations per participant. The NS agent did not appear in this phase. The order within each block was randomized. The CS-US contingency was set at 75%. As in the baseline phase, the startle noise was presented with a contingency of 75%.

The extinction phase consisted of 12 blocks designed in exactly the same way as those in the acquisition phase, except for the absence of the US and the reappearance of the NS agent. Because three agents were presented instead of two, the total number of trials was 36 in this phase. Also in the extinction phase the startle noise was presented with a contingency of 75%. After the baseline, acquisition and extinction phase, a rating phase took place in which each agent was presented (presentation 8 s, inter-stimulus interval 20 s) again without US or startle noise.

In the first session participants were briefed and the informed consent form was signed. After filling out the demographic questionnaire and the SPIN, participants were prepared for the VR part of the experiment. The electrodes, the air blast device, the cuff for the electric stimuli, the HMD and the headphones were adjusted. During the experiment the laboratory room was darkened and participants received recorded instructions via the headphones.

Before the baseline phase started, participants were able to walk around a desk standing in the middle of the room with gray walls and floor in VR. After exploring this virtual environment, the room faded into a gray background and participants relaxed for 2 min in VR. After the baseline phase, participants received the recorded instruction: “You will now meet virtual human beings. Please use the joystick to approach the person. Please try to move directly toward the person. Press the joystick forward to move straight forward and approach the person.” Participants had to approach the agents actively using the joystick and as soon as they reached a specific distance to the agents (the equivalent of about 30 cm in the real world), lights faded out and the next agent was presented at the opposite wall. Each trial lasted about 10 s (depending on how fast participants approached the agents). Theoretically, participants could move laterally, diagonally or away from the agent, however, we observed no such behavior. Because the field of view was adapted to head movements, participants could theoretically look away while moving toward the agent. After the baseline phase, the first rating took place; participants approached each of the three agents and as soon as they reached the previously specified distance to the agents, lights faded out and the participants were asked to verbally rate their subjective fear and the contingency of aversive events.

During the acquisition phase, participants again received the recorded instruction to approach the agents actively via joystick and, as soon as they reached the pre-determined distance to the agents, the lights faded out. At this moment, the US was presented for CS+ agents in 75% of the trials. After the acquisition phase, participants rated the agents again as described above.

The following extinction phase differed from the acquisition only in the reappearance of the NS and the absence of aversive US. After the third rating, the experiment was complete.

### Statistical Analyses

Physiological data were preprocessed with Brain Vision Analyzer 2.0 software (Brain Products GmbH, Munich, Germany) and further analyses were performed in SPSS 22.0 (IBM Corp., Armonk, NY, United States).

For each physiological outcome variable (fear-potentiated startle, SCL) and avoidance behavior, means were calculated for the baseline phase, while the first four reactions and the last four reactions in the acquisition and the extinction phase were computed as the means of the beginning and the end of the acquisition and extinction phase, respectively.

For the fear-potentiated startle, first, differences between the two electromyography electrodes were computed (see Blumenthal et al., 2005). Then, a 250 Hz high cut-off filter, a 30 Hz low cut-off filter, and a 50 Hz notch filter were applied, the data were rectified, and a moving average (50 ms) was calculated. For each fear-potentiated startle a baseline correction was conducted using the mean value of the 50 ms before each startle tone as baseline. Next, peaks were marked automatically, controlled manually and corrected if necessary. Finally, *T*-values for the startle magnitude were calculated. Due to technical errors during data acquisition, six participants had to be excluded from data analysis of the fear-potentiated startle.

For the analysis of the SCL, the difference between the two electrodes was computed, a 1 Hz high cut-off filter and a baseline correction of 1-s duration applied and the SCL exported in order to calculate *T*-values for the SCL. Due to technical errors during data acquisition, five participants had to be excluded from data analysis of the SCL.

The avoidance behavior was assessed via time in non-motion (latency) and time in motion. Time in non-motion (in s) was defined as the time before approaching the agent. Time in motion (in s) was computed subtracting the time in non-motion from the total time needed for reaching the specific distance to the agent.

The means for each agent (CS+, CS-, NS) of the subjective variable (fear and contingency ratings) measured at the three rating phases (rating 1–3) were calculated.

Participants were divided into two groups (low vs. high social anxiety) via a median split of the SPIN score (median = 13.5 in this study) in order to differentiate between highly and less socially fearful participants.

Two repeated-measures ANOVAs with the within-subject factors phase (rating 1 vs. rating 2 for acquisition and rating 2 vs. rating 3 for extinction) and stimulus (CS+ vs. CS- vs. NS) and the between-subject factors social anxiety (low vs. high) and condition (electroshock condition vs. social threat condition) were conducted for both subjective variables.

For each physiological and behavioral outcome variable, repeated-measures ANOVAs with the within-subject factors time (baseline vs. beginning vs. end of acquisition) and stimulus (CS+ vs. CS-) and the between-subject factors social anxiety (low vs. high) and condition (electroshock condition vs. social threat condition) were conducted for the acquisition phase. For the extinction phase repeated-measures ANOVAs with the within-subject factors time (beginning vs. end of extinction) and stimulus (CS+ vs. CS-) and the between-subject factors social anxiety (low vs. high) and condition (electroshock condition vs. social threat condition) were conducted.

Measuring generalization effects, ANOVAs with the within-subject factor phase (baseline vs. end of extinction) and the between-subject factors social anxiety (low vs. high) and condition (electroshock condition vs. social threat condition) were conducted for the NS as well.

In additional analyses of significant effects of time, stimulus, or social anxiety Student’s *t*-tests were performed. Partial η^2^ ($\eta_{p}^{2}$) scores and Cohen’s *d* were used as indices of effect size. The significance level was set at two-tailed alpha = 0.05.

## Results

### Fear Ratings

**FIGURE 3:**
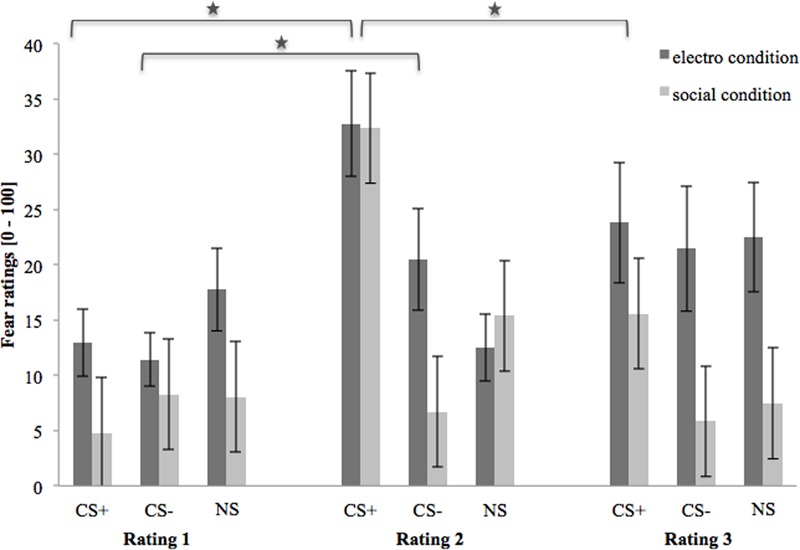
Fear ratings (*n* = 43) for CS+, CS– and NS in the three rating phases for the electro shock and social threat condition. CS+ = agent paired with aversive unconditioned stimulus (US); CS– = agent without aversive US; *NS* = agent without aversive US and not appearing during the acquisition phase; electro condition = electrical stimulation; social condition = air blast combined with virtual spitting and insulting; Rating 1 = after baseline phase; Rating 2 = after acquisition phase; Rating 3 = after extinction phase. Mean fear ratings (0 = very low fear to 100 = very high fear) were given. Significant differences are indicated with an asterisk. Standard errors are presented by error bars.

**Figure 3** shows the fear ratings 1–3 (after the baseline, acquisition and extinction phase, respectively). As we can see, in the beginning, (baseline) fear ratings are almost equal for all three stimuli, but slightly higher in the electroshock than in the social threat condition. After the acquisition phase, fear ratings for CS+ are clearly higher than for CS- and NS in both US conditions. Fear ratings for CS- are higher in the electroshock than in the social threat condition, while fear ratings for NS barely differ after acquisition. After the extinction phase, fear ratings for CS+ decrease in both conditions. However, fear ratings for CS+ decreased more in the social threat condition than in the electroshock condition. CS- did not change in either condition over time, whereas the NS increased in the electroshock condition and decreased in the social threat condition. After extinction, all three stimuli are generally rated with higher fear and contingency levels in the electroshock condition than in the social threat condition.

An ANOVA comparing fear ratings before and after acquisition confirmed significant interaction effects of Phase × Stimulus and Phase × Stimulus × Condition (please see **Table 2** for all significant results of the ANOVA). A follow-up ANOVA was conducted for each condition. For the electroshock condition, a significant interaction effect of Phase × Stimulus could be detected. A follow-up *t*-test showed that the fear ratings increased significantly for CS+, *t*(21) = -5.04, *p* < 0.001, *d* = 1.12, and for CS-, *t*(21) = -2.46, *p* = 0.023, *d* = 0.54, and decreased significantly for NS, *t*(21) = 2.59, *p* = 0.017, *d* = 0.31, from pre to post acquisition. For the social condition, an interaction effect of Phase × Stimulus was also significant. Follow-up *t*-test revealed that fear ratings increased significantly only for CS+, *t*(20) = -5.67, *p* < 0.001, *d* = 1.52, from pre to post acquisition, but not for CS- or NS. Therefore, the fear rating results indicate that successful SFC took place under both conditions.

**Table 2:** Significant results of the ANOVAs for the fear ratings of the acquisition and extinction phase.

Effect	*df*	*F*	η^2^	*p*
**Acquisition**		
Total				
Phase	1, 39	32.1	0.45	<0.001
Stimulus	2, 78	13.9	0.26	<0.001
Phase × Stimulus	2, 78	20.5	0.34	<0.001
Phase × Stimulus × Condition	2, 78	4.96	0.11	0.009
Electroshock condition	
Phase	1, 20	14.4	0.42	<0.001
Stimulus	2, 40	4.72	0.19	0.014
Phase × Stimulus	2, 40	11.8	0.37	<0.001
Social threat condition	
Phase	1, 19	17.6	0.48	<0.001
Stimulus	2, 38	10.2	0.35	<0.001
Time × Stimulus	2, 38	13.1	0.41	<0.001
**Extinction**		
Total				
Stimulus	2, 74	22.7	0.38	<0.001
Phase × Stimulus	2, 74	10.6	0.22	<0.001

**df* = degrees of freedom; η^*2*^ = effect size; *Phase* = Rating 1 vs. Rating 2 for the acquisition and Rating 2 vs. Rating 3 for the extinction; Rating 1 = after baseline, Rating 2 = after acquisition, Rating 3 = after extinction; *Stimulus* = CS+ vs. CS- vs. NS; *CS*+ = agent paired with the aversive unconditioned stimulus (US), CS- = agent without aversive US, *NS* = agent without aversive US and not appearing during the acquisition phase; *Condition* = electroshock vs. social threat condition; *Social Anxiety* (low vs. high) was measured with the German version of the Social Phobia Inventory (SPIN; median split = 13.5, Stangier and Steffens, 2002).*

An ANOVA comparing fear ratings before and after extinction confirmed a significant interaction effect of Phase × Stimulus. Follow-up *t*-test showed that fear ratings decreased significantly for CS+, *t*(40) = 3.92, *p* < 0.001, *d* = 0.60, from pre to post extinction, but not for CS- or NS. The fear rating results indicate that social fear extinction was also successful under both conditions.

### Contingency Ratings

**FIGURE 4:**
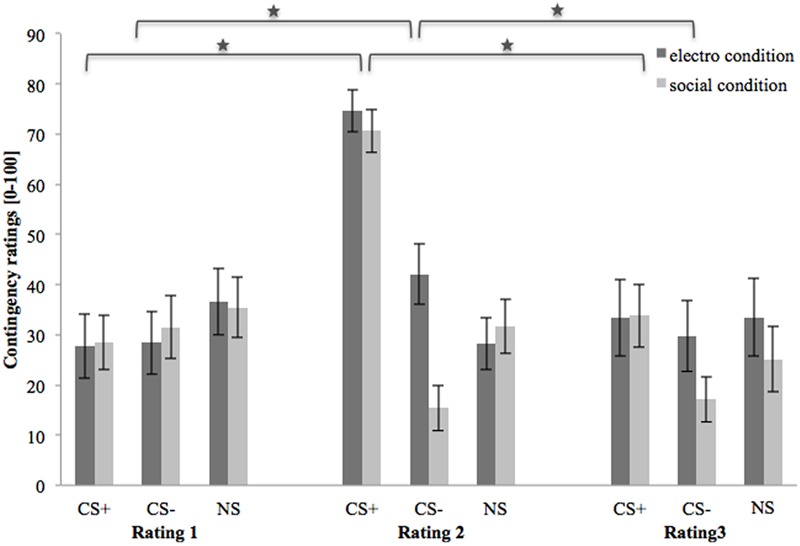
Contingency ratings (n = 43) for CS+, CS– and NS in the three rating phases for the electro shock and social threat condition. CS+ = agent paired with aversive unconditioned stimulus (US); CS– = agent without aversive US; *NS* = agent without aversive US and not appearing during the acquisition phase; electro condition = electrical stimulation; social condition = air blast combined with virtual spitting and insulting; Rating 1 = after baseline phase; Rating 2 = after acquisition phase; Rating 3 = after extinction phase. Mean contingency ratings (0 = very unlikely to 100 = very likely) were given. Significant differences are indicated with an asterisk. Standard errors are presented by error bars.

**Figure 4** shows contingency ratings 1–3 (after baseline, acquisition, and extinction phase, respectively). In the beginning, contingency ratings are almost equal for both conditions and all three stimuli. After the acquisition phase, contingency ratings for CS+ are higher than for CS- or NS in both US conditions. Regarding the CS-, contingency ratings are higher in the electroshock than in the social threat condition. In both conditions the contingency ratings for NS decrease slightly after acquisition. After the extinction phase, the contingency ratings for CS+ decrease strongly in both conditions. Contingency ratings for CS- decrease in the electroshock condition and increase slightly in the social threat condition. Conversely, contingency ratings for NS increased slightly in the electroshock condition and decreased slightly in the social threat condition.

An ANOVA comparing contingency ratings before and after acquisition confirmed significant interaction effects of Phase × Stimulus, Stimulus × Social Anxiety, and Phase × Stimulus × Condition (please see **Table 3** for all significant results of the ANOVA). Follow-up ANOVA was conducted for each condition. In the electroshock condition, significant interaction effects of Phase × Stimulus, and Stimulus × Social Anxiety could be detected. Follow-up *t*-test conducted for Phase × Stimulus interaction showed that contingency ratings increased significantly for CS+, *t*(21) = -7.49, *p* < 0.001, *d* = 1.88, and for CS-, *t*(21) = -2.38, *p* = 0.027, *d* = 0.48, from pre to post acquisition, but not for NS. Follow-up tests of the significant Stimulus × Social Anxiety interaction revealed a significant difference for the less socially fearful participants between CS+, CS-, and NS (*p* < 0.020), and for the higher socially fearful participants between CS+ and CS- (*p* < 0.003), but not NS. Means and standard deviations are presented in **Table 4**. In the social threat condition an interaction effect of Phase × Stimulus reached significance level. Follow-up *t*-test showed that contingency ratings increased significantly for CS+, *t*(19) = -7.50, *p* < 0.001, *d* = 1.88, and decreased for CS-, *t*(19) = 2.47, *p* = 0.023, *d* = 0.72, from pre to post acquisition. This pattern could not be found for NS. Thus, contingency rating results also indicate that SFC was successful.

**Table 3:** Significant results of the ANOVAs for the contingency ratings of the acquisition and the extinction phase.

Effect	*df*	*F*	η^2^	*p*
**Acquisition**		
Total		
Phase	1, 38	10.8	0.22	0.002
Stimulus	2, 76	33.9	0.47	<0.001
Phase × Stimulus	2, 76	51.3	0.58	<0.001
Stimulus × Social Anxiety	2, 76	3.29	0.08	0.042
Phase × Stimulus × Condition	2, 76	5.76	0.13	0.005
Electroshock condition	
Phase	1, 20	15.5	0.44	<0.001
Stimulus	2, 40	10.8	0.35	<0.001
Phase × Stimulus	2, 40	25.4	0.56	<0.001
Stimulus × Social Anxiety	2, 40	3.98	0.17	0.027
Social threat condition	
Stimulus	2, 36	28.0	0.61	<0.001
Phase × Stimulus	2, 36	30.9	0.63	<0.001
**Extinction**		
Total		
Phase	1, 37	13.6	0.27	<0.001
Stimulus	2, 74	71.5	0.66	<0.001
Stimulus × Condition	2, 74	8.04	0.18	<0.001
Stimulus × Social Anxiety	2, 74	6.72	0.15	0.002
Phase x Stimulus	2, 74	31.9	0.46	<0.001
Phase × Stimulus × Condition	2, 74	3.06	0.08	0.053
Electroshock condition	
Phase	1, 19	6.49	0.26	0.020
Stimulus	2, 38	26.0	0.58	<0.001
Phase × Stimulus	2, 38	20.2	0.52	<0.001
Stimulus × Social Anxiety	2, 38	4.30	0.19	0.021
Social threat condition	
Phase	1, 18	7.71	0.30	0.012
Stimulus	2, 36	49.3	0.73	<0.001
Phase × Stimulus	2, 36	14.9	0.45	<0.001
Stimulus × Social Anxiety	2, 36	4.51	0.20	0.018

*df = degrees of freedom; η^*2*^ = effect size; *Phase* = Rating 1 vs. Rating 2 for the acquisition and Rating 2 vs. Rating 3 for the extinction; Rating 1 = after baseline, Rating 2 = after acquisition, Rating 3 = after extinction; *Stimulus* = CS+ vs. CS- vs. NS; CS+ = agent paired with the aversive unconditioned stimulus (US), CS- = agent without aversive US, *NS* = agent without aversive US and not appearing during the acquisition phase; *Condition* = electroshock vs. social threat condition; *Social Anxiety* (low vs. high) was measured with the German version of the Social Phobia Inventory (SPIN; median split = 13.5, Stangier and Steffens, 2002).*

**Table 4:** Means (M) and standard deviations (SD) for contingency ratings during acquisition and extinction for high- and low-social anxious and both conditions.

	CS+		CS-		NS	
	*M*	*SD*	*M*	*SD*	*M*	*SD*
**Acquisition**		
*Electroshock condition*						
Low socially fear	49.9	22.5	35.4	30.5	24.6	22.4
High socially fear	53.4	18.8	35.0	15.3	45.9	24.2
**Extinction**						
*Electroshock condition*						
Low socially fear	51.7	22.2	33.9	28.6	23.4	19.3
High socially fear	57.2	22.3	38.4	28.1	43.3	21.8
*Social threat condition*						
Low socially fear	63.6	13.9	15.6	19.4	27.5	22.4
High socially fear	44.6	19.5	16.7	19.7	29.1	17.3

*CS+ = agent paired with US, CS- = agent without aversive US, NS = agent without aversive US and not appearing during the acquisition phase; Social Anxiety (low vs. high) was measured with the German version of the Social Phobia Inventory (SPIN; median split = 13.5, Stangier and Steffens, 2002).*

An ANOVA on contingency ratings before and after extinction showed significant interaction effects for Stimulus × Condition, Stimulus × Social Anxiety, Phase × Stimulus, and a marginally significant interaction effect of Phase × Stimulus × Condition. Follow-up ANOVAs were conducted separately for the two conditions. In the electroshock condition, interaction effects of Phase × Stimulus and Stimulus × Social Anxiety reached significance level. Follow-up *t*-test conducted for the Phase × Stimulus interaction effect showed that contingency ratings decreased significantly for CS+, *t*(20) = 5.88, *p* < 0.001, *d* = 1.66, and for CS-, *t*(20) = 2.66, *p* = 0.015, *d* = 0.46, from pre to post extinction, but not for NS. Follow-up tests of the Stimulus × Social Anxiety interaction revealed a significant difference both for the less socially fearful participants between CS+ and NS (*p* < 0.020), and for the highly socially fearful participants between CS+, CS- and NS (*p* < 0.022). In the social threat condition, interaction effects of Phase × Stimulus and Stimulus × Social Anxiety reached significance level. Follow-up *t*-tests of the Phase × Stimulus interaction revealed that contingency ratings decreased significantly for CS+, *t*(19) = 5.91, *p* < 0.001, *d* = 1.58, but not for CS- or NS. Follow-up tests of the significant Stimulus × Social Anxiety interaction revealed a significant difference both for the less socially fearful participants between CS+, CS- and NS (*p* < 0.001), and for the highly socially fearful participants between CS+, CS- and NS (*p* < 0.030). These results indicate that social fear extinction was successful according to the contingency ratings as well.

### Fear-Potentiated Startle

**FIGURE 5:**
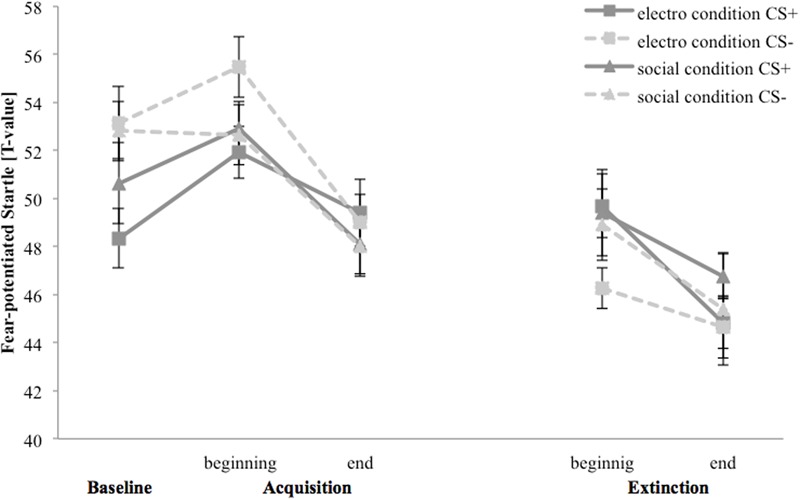
Fear-potentiated startle response (*n* = 37) for CS+ and CS– in the three phases (baseline, acquisition, and extinction) for the electro shock and social threat condition. CS+ = agent paired with aversive unconditioned stimulus (US); CS– = agent without aversive US; electro condition = electrical stimulation; social condition = air blast combined with virtual spitting and insulting. Mean fear-potentiated startles (presented in *T*-values) was given. Standard errors are presented by error bars.

**Figure 5** depicts fear-potentiated startle response for the baseline, acquisition and extinction phase. In the electroshock condition fear-potentiated startle response is higher for CS- than for CS+ at the baseline and both stimuli increase at the beginning, until both decrease to the end of the acquisition. In the extinction phase CS+ response is higher than CS-, but the responses to both stimuli decreased from the beginning to the end. In the social threat condition fear-potentiated startle response is higher for CS- than for CS+ at the baseline. CS+ response increases whereby CS- do not change at the beginning, until both decrease at the end of the acquisition. In the extinction phase both stimuli decrease from the beginning to the end.

For the acquisition phase, an ANOVA confirmed a significant main effect of time, *F*(1,33) = 7.51, *p* < 0.001, $\eta_{p}^{2}$ = 0.19, and stimulus, *F*(1,33) = 5.20, *p* = 0.029, $\eta_{p}^{2}$ = 0.14, but no significant interaction effects. **Figure 5** shows an increase of fear-potentiated startle at the beginning and a fast habituation process at the end of the acquisition phase in both conditions.

For the extinction phase, there was a significant main effect of time, *F*(1,31) = 8.46, *p* = 0.007, $\eta_{p}^{2}$ = 0.21, but no other significant main or interaction effects. For NS, a significant main effect of time, *F*(1,32) = 7.98, *p* = 0.008, $\eta_{p}^{2}$ = 0.20, could be detected.

### Skin Conductance Level

**FIGURE 6:**
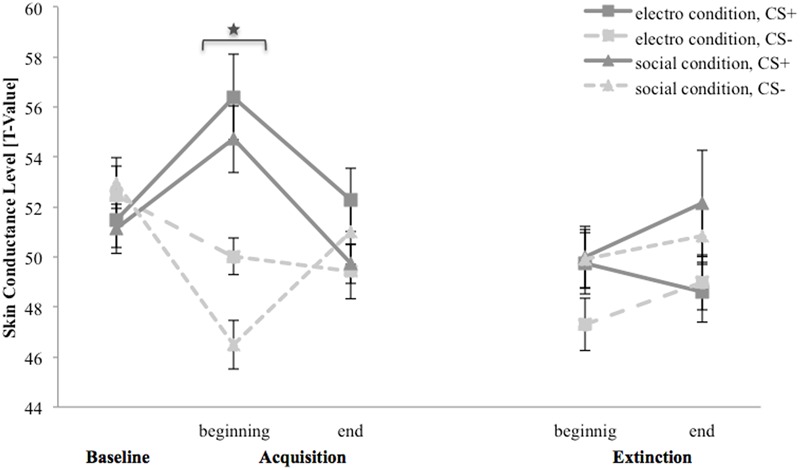
Skin conductance level (*n* = 38) for CS+ and CS– in the three phases (baseline, acquisition, and extinction) for the electro shock and social threat condition. CS+ = agent paired with aversive unconditioned stimulus (US); CS– = agent without aversive US; electro condition = electrical stimulation; social condition = air blast combined with virtual spitting and insulting. Mean skin conductance level (presented in *T*-values) was given. Significant differences are indicated with an asterisk. Standard errors are presented by error bars.

**Figure 6** depicts SCL for the baseline, acquisition and extinction phase. In the baseline, SCL for CS+ response is slightly higher than for CS- in both conditions. In the electroshock condition, for CS+ the SCL increase from the baseline to the beginning and decrease to the end of the acquisition, whereas it decrease for CS- from the baseline to the end of the acquisition. In the beginning of the extinction, SCL for CS+ is higher than for CS-, at the end of the extinction both stimuli do not differ. In the social condition, SCL for CS+ also increase from the baseline to the beginning and decrease from the beginning to the end of the acquisition. SCL for CS- decrease from the baseline to the beginning and subsequently increase to the end of the acquisition. In the beginning of the extinction, both stimuli do not differ and both increase slightly at the end of the extinction.

For the acquisition phase, an ANOVA confirmed significant main effects of stimulus, *F*(1,34) = 15.4, *p* = 0.010, $\eta_{p}^{2}$ = 0.18, as well as significant interaction effect of Time × Stimulus, *F*(2,68) = 18.5, *p* < 0.001, $\eta_{p}^{2}$ = 0.35. Follow-up *t*-tests revealed that SCL for CS+ and CS- only differed at the beginning of the acquisition, *t*(37) = 6.26, *p* < 0.001, *d* = 1.35. Thus, there was a significant increase in SCL for CS+ and a significant decrease for CS- from the baseline to the beginning of the acquisition. The SCL results indicate that successful SFC took place under both condition, but also a fast habituation during acquisition.

For the extinction phase, an ANOVA showed a significant main effect of condition, *F*(1,32) = 4.95, *p* = 0.033, $\eta_{p}^{2}$ = 0.13, and a significant interaction effect of Time × Stimulus × Condition × Social Anxiety, *F*(1,32) = 101.8, *p* = 0.044, $\eta_{p}^{2}$ = 0.12. A follow-up ANOVA was conducted separately for both conditions. In the electroshock condition, no significant main or interaction effects were found. In the social threat condition, a significant interaction effect of Time × Stimulus × Social Anxiety, *F*(1,17) = 4.48, *p* = 0.049, $\eta_{p}^{2}$ = 0.21, was detected. Follow-up *t*-tests conducted separately for higher and less socially fearful participants neither showed significant differences between SCL for CS+ and CS- at the beginning nor at the end of the extinction. For NS, a significant main effect of time, *F*(1,33) = 7.39, *p* = 0.010, $\eta_{p}^{2}$ = 0.18, could be detected.

### Avoidance (Time in Non-motion)

**FIGURE 7:**
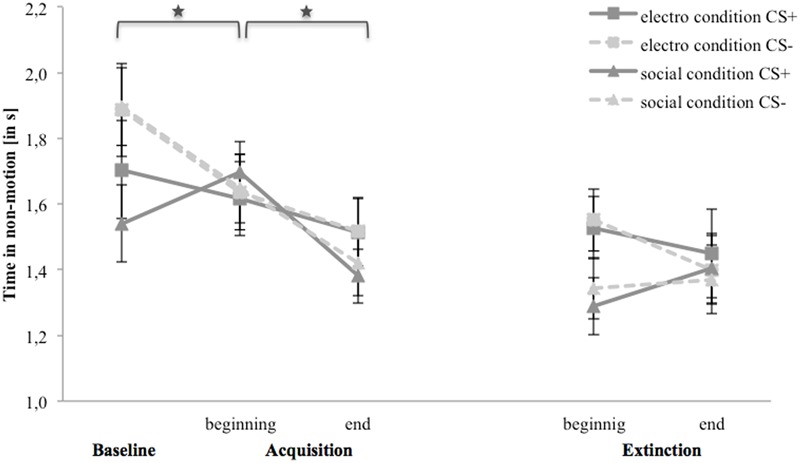
Time in non-motion (*n* = 36) for CS+ and CS– in the three phases (baseline, acquisition, and extinction) for the electro shock and social threat condition. CS+ = agent paired with aversive unconditioned stimulus (US); CS– = agent without aversive US; electro condition = electrical stimulation; social condition = air blast combined with virtual spitting and insulting. Mean time in non-motion (in s) was given. Significant differences are indicated with an asterisk. Standard errors are presented by error bars.

**Figure 7** shows time in non-motion for the baseline, acquisition and extinction phase. In the electroshock condition, avoidance for both stimuli decreases from the baseline to the end of the acquisition phase as well as from the beginning to the end of the extinction phase. In the social threat condition, avoidance for CS- is higher than for CS+ at the baseline, and to the end of the acquisition phase it decreases for CS-, whereas avoidance increases for CS+ from the baseline to the beginning until it decreases at the end of the acquisition. In the extinction phase, both stimuli do not differ at any point.

For the acquisition phase, an ANOVA confirmed significant interaction effects of Time × Stimulus, and Condition × Social Anxiety (please see **Table 5** for all significant results of the ANOVA). Follow-up ANOVA was conducted separately for both conditions. In the electroshock condition, no significant interaction effects were found. In the social threat condition, a significant interaction effect of Time × Stimulus could be detected. Follow-up *t*-tests showed that avoidance for CS+ increased from the baseline to the beginning of the acquisition phase, *t*(18) = -2.13, *p* = 0.047, *d* = 0.33, and decreased from the beginning to the end of the acquisition phase, *t*(18) = 3.32, *p* = 0.004, *d* = 0.84. Avoidance for CS- decreased from the baseline to the beginning of the acquisition, *t*(18) = 2.35, *p* = 0.031, *d* = 0.53, as well as from the beginning to the end of the acquisition, *t*(18) = 2.77, *p* = 0.013, *d* = 0.51. Therefore, the time in non-motion results indicate that successful avoidance behavior for CS+ took place in the social threat condition, but also a fast adaptation to the US occurred toward the end of the acquisition.

**Table 5:** Significant results of the ANOVAs for avoidance (time in non-motion) of the acquisition and extinction phase.

Effect	*df*	*F*	η^2^	*p*
**Acquisition**		
Total				
Time	2, 64	10.5	0.25	<0.001
Stimulus	1, 32	9.83	0.24	0.004
Time x Stimulus	2, 64	9.34	0.23	<0.001
Condition × Social Anxiety	1, 32	5.42	0.15	0.026
Electroshock condition				
Time	2, 30	4.06	0.21	0.027
Stimulus	1, 15	6.71	0.31	0.020
Social threat condition				
Time	2, 34	7.82	0.32	0.002
Stimulus	1, 17	5.27	0.24	0.035
Time × Stimulus	2, 34	7.02	0.29	0.003
**Extinction**		
*Total*				
Social Anxiety	1, 32	4.71	0.13	0.038
Time × Condition	1, 32	4.25	0.12	0.047
Electroshock condition				
Social Anxiety	1, 15	9.56	0.39	0.007

*df = degrees of freedom; η^*2*^ = effect size; *Phase* = Rating 1 vs. Rating 2 for the acquisition and Rating 2 vs. Rating 3 for the extinction; Rating 1 = after baseline, Rating 2 = after acquisition, Rating 3 = after extinction; *Stimulus* = CS+ vs. CS- vs. NS; CS+ = agent paired with the aversive unconditioned stimulus (US), CS- = agent without aversive US, *NS* = agent without aversive US and not appearing during the acquisition phase; *Condition* = electroshock vs. social threat condition; *Social Anxiety* (low vs. high) was measured with the German version of the Social Phobia Inventory (SPIN; median split = 13.5, Stangier and Steffens, 2002).*

For the extinction phase, an ANOVA confirmed a significant interaction effect of Time × Condition. Follow-up ANOVA was conducted separately for both conditions. In the electroshock condition, only a significant main effect of social anxiety was found. In the social threat condition, no significant effects were found. For NS, a significant main effect of time, *F*(1,32) = 4.81, *p* = 0.036, $\eta_{p}^{2}$ = 0.13, could be detected.

### Avoidance (Time in Motion)

**FIGURE 8:**
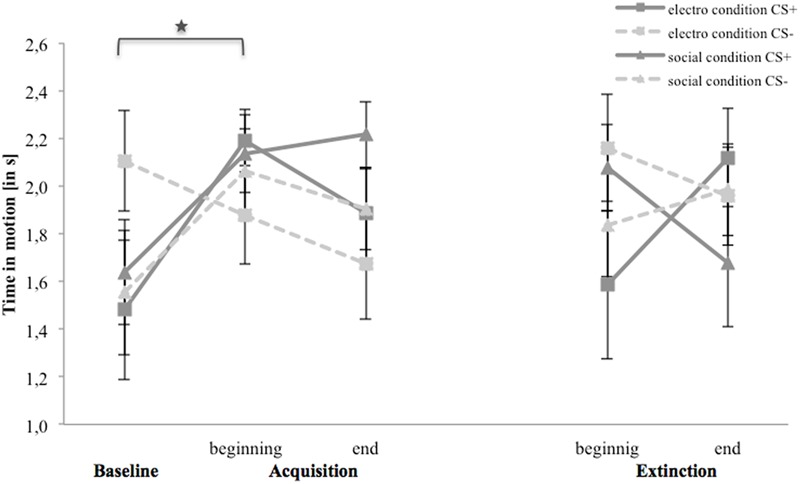
Time in motion (*n* = 36) for CS+ and CS– in the three phases (baseline, acquisition, and extinction) for the electro shock and social threat condition. CS+ = agent paired with aversive unconditioned stimulus (US); CS– = agent without aversive US; electro condition = electrical stimulation; social condition = air blast combined with virtual spitting and insulting. Mean time in non-motion (in s) was given. Significant differences are indicated with an asterisk. Standard errors are presented by error bars.

**Figure 8** shows time in motion for the baseline, acquisition and extinction phase. In the electroshock condition, the avoidance of CS- is higher than of CS+ during the baseline. Avoidance toward CS- decreases from the baseline to the end of the acquisition, whereas it increases for CS+ from the baseline to the beginning and decreases to the end of the acquisition. In the extinction phase participants move faster toward CS- and slower toward CS+ from the beginning to the end of the extinction. In the social threat condition, time to approach both stimuli are equally long during baseline and increase at the beginning of the acquisition, until avoidance to both stimuli stay approximately at the same level at the end of the acquisition. In the extinction phase, the avoidance of CS+ decreases during the extinction, whereas for CS- it stays on an equal level.

For the acquisition phase, an ANOVA confirmed significant interaction effects of Time × Stimulus and Condition × Social Anxiety (please see **Table 6** for all significant results of the ANOVA). Follow-up ANOVAs were conducted separately for both conditions. In the electroshock condition, a significant interaction effect of Time × Stimulus could be detected. Follow-up *t*-tests revealed that only the CS+ significantly increased from the baseline to the beginning of the acquisition, *t*(37) = -2.45, *p* = 0.026, *d* = 0.77. In the social threat condition, no significant interaction effects were found. Therefore, time in motion results indicate a successful SFC at the beginning of the acquisition in the electroshock condition, but also a fast adaptation to the US occurred toward the end of the acquisition.

**Table 6:** Significant results of the ANOVAs for avoidance (time in motion) of the acquisition and extinction phase.

Effect	*df*	*F*	η^2^	*p*
**Acquisition**		
Total				
Time	2, 64	139.8	0.81	<0.001
Social Anxiety	1, 32	7.07	0.18	0.012
Time × Stimulus	2, 64	4.68	0.13	0.013
Condition × Social Anxiety	1, 32	6.37	0.17	0.017
Electroshock condition				
Time	2, 30	75.1	0.83	<0.001
Stimulus	1, 15	5.60	0.27	0.032
Social Anxiety	1, 15	9.18	0.38	0.008
Time × Stimulus	2, 30	4.67	0.24	0.017
Social threat condition				
Time	2, 30	5.10	0.25	0.039
**Extinction**		
Total				
Time × Stimulus × Condition	1, 32	5.87	0.16	0.021
Time × Stimulus × Condition × Social Anxiety	1, 32	6.45	0.17	0.016
Electroshock condition				
Time × Stimulus	1, 15	5.02	0.25	0.041
Time × Stimulus × Social Anxiety	1, 15	6.91	0.32	0.019

*df = degrees of freedom; η^*2*^ = effect size; *Phase* = Rating 1 vs. Rating 2 for the acquisition and Rating 2 vs. Rating 3 for the extinction; Rating 1 = after baseline, Rating 2 = after acquisition, Rating 3 = after extinction; *Stimulus* = CS+ vs. CS- vs. NS; CS+ = agent paired with the aversive unconditioned stimulus (US), CS- = agent without aversive US, *NS* = agent without aversive US and not appearing during the acquisition phase; *Condition* = electroshock vs. social threat condition; *Social Anxiety* (low vs. high) was measured with the German version of the Social Phobia Inventory (SPIN; median split = 13.5, Stangier and Steffens, 2002).*

For the extinction phase, an ANOVA confirmed significant interaction effects of Time × Stimulus × Condition, and Time × Stimulus × Condition × Social Anxiety. Follow-up ANOVA for the electroshock condition revealed a significant interaction effect of Time × Stimulus, and Time × Stimulus × Social Anxiety. Further follow-up ANOVAs were conducted separately for the low and high social fear groups, but no significant main or interaction effects were found. No significant effects were found in the social threat condition or for the NS.

## Discussion

The aim of this study was to replicate and extend the findings of our previous study we conducted on social fear learning (Shiban et al., 2015). In order to improve the paradigm, we investigated the “belongingness effect” (Hamm et al., 1989). To this end, we designed a social threat condition and compared it to an electroshock condition during the different phases (baseline, acquisition and extinction) of the social fear conditioning paradigm (SFC). Participants actively approached virtual agents using a joystick in a setting similar to the one used by Shiban et al. (2015). Social fear learning was examined via subjective ratings (fear and contingency ratings), physiological (fear-potentiated startle, skin conductance level) and behavioral measures (avoidance).

Social fear acquisition was successful according to the fear and the contingency ratings. In both conditions, these measures clearly increased for CS+ compared to CS- from the baseline to the end of the acquisition phase. Interestingly, there was a higher differentiation between CS+ and CS- in the social threat compared to the electroshock condition, which might reflect a tendency toward higher belongingness in the social threat condition. Regarding the physiological outcome variables, the fear-potentiated startle results did not confirm our hypotheses, as no discrimination between CS+ and CS- could be detected. However, with respect to the SCL, successful fear conditioning took place at the beginning of the acquisition, whereas a fast habituation was found toward the end of acquisition, diminishing any discriminant effects between the CS+ and CS-. Furthermore, the avoidance behavior clearly increased for CS+ compared to CS- at the beginning of the acquisition phase for the time in non-motion in the social threat condition and the time in motion in the electroshock condition.

Fear extinction was evident in the ratings, as the differentiation in terms of fear and contingency ratings between the CS+ and the CS- that followed acquisition vanished during the extinction phase for both experimental groups. However, no statistically significant extinction was found in the physiological and behavioral variables. It is possible that the physiological level had already been subject to a fast extinction process that can be expected in non-socially phobic individuals before the designated extinction phase of the experiment.

According to our data, social fear can be induced and extinguished confirming the operant conditioning paradigm. Participants did not simply explore the virtual room and the agents in our (operant) fear conditioning paradigm, but actively (using a joystick) approached the agents. They were free to decide how fast they wanted to approach the agents and to which degree they wanted to avoid them. With participants being punished while approaching the stimuli (virtual male agents), our SFC paradigm reflects operant conditioning rather than classical conditioning processes. Interestingly, less socially fearful participants differentially evaluated the contingency of CS+, CS-, and NS after extinction in the electroshock condition and only rated the contingency of the CS+ as high, whereas higher socially fearful participants rated the contingency of the CS+ and the NS on a similar level. Thus, we found a generalization effect in the contingency ratings between CS+ and NS for higher socially fearful participants. No generalization effect was reflected by the physiological measures.

Summarizing the results for the subjective ratings as well as the physiological and behavioral data, our initial hypotheses could be partially confirmed. The habituation at the end of the acquisition phase might reflect a fast adaptation to the aversive US. Possibly the US was not aversive enough to evoke long-lasting fear or the social anxiety of the sample was too low. Due to the belongingness effect, a higher differentiation in the subjective ratings between CS+ and CS- in the social threat condition was found.

Our SFC paradigm might have induced an approach-avoidance conflict. This conflict occurs when a person is faced with the decision to either pursue or avoid something that is advantageous in some respects but disadvantageous in others. In the social threat condition, the avoidance behavior (time in non-motion) clearly differed between aversive (CS+) and non-aversive (CS-) stimuli at the beginning of the acquisition. By comparison, in the electroshock condition the avoidance behavior (time in motion) clearly increased toward aversive compared to non-aversive stimuli at the beginning of the acquisition. Avoiding social situations is a core feature of SAD. Our paradigm showed increased fear and a partial increase in avoidance after the presentation of the first four aversive agents during conditioning. Besides behavioral avoidance, eye-gaze, a non-verbal social cue, is an important aspect of human social behavior. Future studies may therefore consider measuring behavioral approach-avoidance conflict via an eye-tracking method and analyze the recorded movement trajectories as an index of avoidance behavior for social anxiety. Identifying approach- and avoidance-related responses to social stimuli like emotional face stimuli (e.g., via reaction times for pressing a button or joystick responses, or through eye-gaze), has already been investigated in different studies (Mühlberger et al., 2008; Wieser et al., 2009, 2010; Radke et al., 2013). Wieser et al. (2010), e.g., reported that high anxiety was related to less gaze contact and greater backward head movement in response to male virtual agents, which showed a direct gaze. Furthermore, Dechant et al. (2017) revealed that highly fearful participants showed more avoidance in a social fear virtual paradigm than low fearful participants. It should be noted that avoidance behavior is a crucial element not only in fear learning but also in the maintenance of fear. In this study, we only focused on the fear learning process. In order to investigate the mechanisms of avoidance behavior in SAD in its entirety, we recommend future research to also study the role of safety behaviors in the maintenance of SAD.

In past studies using stimuli of low ecological validity with regard to the nature of SAD, it remained unclear whether socially fearful persons react more sensitively to socially relevant stimuli. Our social threat condition utilizes social stimuli, which are likely to be disorder-relevant for SAD. Thus, our social threat condition might be more suitable for investigating social anxiety due to a higher belongingness between the CS and the US and consequently an enhanced ecological validity of the design. Furthermore, not using electric shocks may make the recruitment of clinical samples easier for future studies. Empirical findings indicate that successful conditioning in highly fearful individuals cannot only be induced by effective non-social US (i.e., electric shocks), but also by social stimuli, such as emotional facial expressions paired with compatible verbal feedback (Lissek et al., 2008) or isolated verbal comments (Ahrens et al., 2014). In the present study, conditioning was successful and avoidance behavior could be observed in both conditions. Still, there was a better differentiation between aversive and non-aversive stimuli in the social threat condition. One explanation for not having observed an enhanced belongingness effect in our study could be that the high social anxiety group showed a low SPIN score (median score = 13.5) as well. According to Connor et al. (2000) a SPIN score of 19 distinguishes between social phobia subjects and controls.

It is noteworthy that participants undergoing electrical stimulation typically have a more robust fear response both before and after acquisition and extinction (Schmitz and Grillon, 2012) and rate the shock as more aversive than alternative stimuli such as a female scream (Glenn et al., 2012), suggesting that they tend to overestimate the probability of aversive stimuli when being physically harmed. However, this effect could not be found in the contingency ratings, and although the subjective fear ratings before acquisition were generally higher for subjects in the electroshock condition, the fear ratings for the CS+ after acquisition barely differed. Furthermore, we found a better differentiation between the CS+ and the CS- both after the acquisition and the extinction phases in the social threat condition than in the electroshock condition, indicating that the social threat is more realistic than the electroshock condition. These findings partially confirm our hypothesis that acquisition and resistance to extinction are intensified by a sense of belongingness between the CS and the applied US. This is an important fact which should be taken into consideration in future research.

An issue regarding the experimental setting is the linguistic label of the fear ratings. Many subjects reported that it was not actually fear they had experienced, but a feeling comparable with unpleasantness or, especially in the case of the virtual spitting, even disgust. Being spat at might not only induce social fear (as expected for a socially fearful person) but also cause disgust. Still, being spat at along with hearing the agent say “go away” is a social situation that is expected to elicit emotions similar to the ones induced in a social fearful or phobic patient. In order to investigate if conditioning had caused social fear or simply disgust, we could have asked participants which emotions had been elicited by the conditioning paradigm. Updating the understanding of SAD, future studies should measure disgust and similar emotions. Furthermore, it has to be taken into account that the three virtual agents differed in clothing, hair color and facial design, which might have led to an association of the US with the external stimuli instead of the situation. As a further limitation of the current study, our non-clinical sample was limited to young students with a high proportion of female students, which should be taken into account when generalizing the results to a broader population. However, as social phobia is twice as prevalent in women than in men, females are an interesting target group for our paradigm (Bandelow and Wedekind, 2014).

Despite these facts, all in all our paradigm has been shown to be suitable for investigating the acquisition and extinction of social fear in a VR setting similar to the paradigm used by Shiban et al. (2015). As in this previous work, results support the translation of the SFC paradigm by Toth et al. (2012) from the mice model to human studies. Further research is needed to expand these findings by increasing the sample size and by testing patients suffering from social phobia. Treatment for this widespread health issue could potentially be enhanced by optimizing the extinction process that is strived for in exposure therapy. Furthermore, it is an interesting research question if patients suffering from social phobia could benefit from extinction processes in different contexts as Dunsmoor et al. (2014) could verify for healthy humans.

## Ethics Statement

This study was carried out in accordance with the recommendations of the Ethics Committee of the University of Regensburg with written informed consent from all subjects. All subjects gave written informed consent in accordance with the Declaration of Helsinki. The protocol was approved by the Ethics Committee of the University of Regensburg.

## Author Contributions

JR study conception, data analysis, wrote the manuscript. SP study conception, data acquisition and analysis, and contribution to the manuscript. JW and VZ data analysis, contribution to the manuscript. YS study conception, data analysis, contribution to the manuscript. All authors have approved of the final version of the manuscript and its submission.

## Conflict of Interest Statement

The authors declare that the research was conducted in the absence of any commercial or financial relationships that could be construed as a potential conflict of interest. The reviewer IN declared a shared affiliation, though no other collaboration, with the authors to the handling Editor.
